# Behavioural Activation Therapy for Depression after Stroke (BEADS): a study protocol for a feasibility randomised controlled pilot trial of a psychological intervention for post-stroke depression

**DOI:** 10.1186/s40814-016-0072-0

**Published:** 2016-08-10

**Authors:** Shirley A. Thomas, Elizabeth Coates, Roshan das Nair, Nadina B. Lincoln, Cindy Cooper, Rebecca Palmer, Stephen J. Walters, Nicholas R. Latimer, Timothy J. England, Laura Mandefield, Timothy Chater, Patrick Callaghan, Avril E. R. Drummond

**Affiliations:** 1 Division of Rehabilitation and Ageing, School of Medicine, B Floor Medical School, Queens Medical Centre, University of Nottingham, Nottingham, NG7 2UH UK; 2 Sheffield Clinical Trials Research Unit, School of Health and Related Research, University of Sheffield, Regent Court, 30 Regent Street, Sheffield, S1 4DA UK; 3 School of Health and Related Research, University of Sheffield, Regent Court, 30 Regent Street, Sheffield, S1 4DA UK; 4 Vascular Medicine, Division of Medical Sciences and GEM, School of Medicine, Royal Derby Hospital, University of Nottingham, Uttoxeter Road, Derby, DE22 3DT UK; 5 School of Health Sciences, A Floor, South Block, Queens Medical Centre, University of Nottingham, Nottingham, NG7 2HA UK

**Keywords:** Stroke, Depression, Post-stroke depression, Psychological intervention, Behavioural activation, Feasibility, Randomised controlled trial, Complex intervention

## Abstract

**Background:**

There is currently insufficient evidence for the clinical and cost-effectiveness of psychological therapies for treating post-stroke depression.

**Methods/Design:**

BEADS is a parallel group feasibility multicentre randomised controlled trial with nested qualitative research and economic evaluation. The aim is to evaluate the feasibility of undertaking a full trial comparing behavioural activation (BA) to usual stroke care for 4 months for patients with post-stroke depression. We aim to recruit 72 patients with post-stroke depression over 12 months at three centres, with patients identified from the National Health Service (NHS) community and acute services and from the voluntary sector. They will be randomly allocated to receive behavioural activation in addition to usual care or usual care alone. Outcomes will be measured at 6 months after randomisation for both participants and their carers, to determine their effectiveness. The primary clinical outcome measure for the full trial will be the Patient Health Questionnaire-9 (PHQ-9). Rates of consent, recruitment and follow-up by centre and randomised group will be reported. The acceptability of the intervention to patients, their carers and therapists will also be assessed using qualitative interviews. The economic evaluation will be undertaken from the National Health Service and personal social service perspective, with a supplementary analysis from the societal perspective. A value of information analysis will be completed to identify the areas in which future research will be most valuable.

**Discussion:**

The feasibility outcomes from this trial will provide the data needed to inform the design of a definitive multicentre randomised controlled trial evaluating the clinical and cost-effectiveness of behavioural activation for treating post-stroke depression.

**Trial registration:**

Current controlled trials ISRCTN12715175

## Background

Depression is the most commonly investigated emotional consequence of stroke [1] with an average prevalence of 29 %, which remains consistent up to 10 years post-stroke [2]. Effective treatment of depression is important because depression is associated with increased healthcare utilisation [3], worse rehabilitation outcomes [4–7], increased carer strain [8] and increased mortality [2, 9]. Co-morbid long-term physical health conditions and mental health problems have been found to increase health care costs [10]. Stroke patients who are depressed may engage less in rehabilitation, which in turn can lead to decreased functional recovery [7]. Post-stroke depression is also associated with lower quality of life [2, 11]. Thus, in addition to improving mood, effective treatment of post-stroke depression is important because it has the potential to improve patients’ functional outcomes and quality of life and also reduce strain on their carers. About one third of stroke patients have aphasia [12, 13] and approximately 70 % will have cognitive impairment [14, 15]. Aphasia can affect all communication modalities including speaking, understanding, reading and writing. Stroke survivors with aphasia may be particularly susceptible to post-stroke depression [16, 17].

There is currently insufficient evidence for the clinical and cost-effectiveness of psychological therapies for treating post-stroke depression [18]. Trials of brief psychosocial behavioural intervention plus antidepressant [19] and motivational interviewing [20, 21] reduced post-stroke depression but these studies recruited patients early after stroke and excluded those with severe communication or cognitive problems, so these findings may not be applicable to all patients with post-stroke depression. There is evidence from single-case design studies that some patients with post-stroke depression improve following cognitive behavioural therapy (CBT) [22, 23]. However, the only randomised controlled trial of CBT for post-stroke depression found no significant difference between those patients who received CBT, an attention placebo or usual care [24].

A psychological intervention which may be suitable for stroke patients is behavioural activation (BA) therapy. BA is based on the behavioural model of depression, where depression is believed to result from a lack of response-contingent positive reinforcement [25]. Positive reinforcement is dependent on the person’s actions [26] and reduction in activity can lead to loss of reinforcement. Stroke can result in a loss or restriction of rewarding activities and interactions (such as everyday activities, hobbies and social interactions) and this loss may lead to depression. BA aims to increase activity level, particularly the frequency of pleasant events, to improve mood. BA is effective at treating depression in adults in primary care settings, older adults and patient/carer dyads with dementia, and has comparable effectiveness to CBT [27–32].

A multicentre randomised controlled trial, the Communication and Low Mood: CALM trial [33] (*n* = 105) evaluated BA, delivered by an assistant psychologist, for treating low mood in stroke patients with aphasia. This found that mood was significantly better at 6-month follow-up in those who received BA compared to usual clinical care. The transferability of BA to hard-to-reach populations, such as those with aphasia and severe cognitive problems [34, 35], adds to its potential as a psychological intervention after stroke. Given that the CALM trial demonstrated that it was possible to deliver BA to a group of patients usually excluded from psychological interventions, there is significant potential for using BA for treating all stroke patients with depression.

However, as the CALM trial was not conducted with the wider stroke population, a more robust pilot study is now required to inform any future proposal for a definitive multicentre randomised controlled trial (RCT) evaluating the clinical and cost-effectiveness of BA for treating post-stroke depression [36–38]. Therefore, the BEADS trial is looking at the feasibility of delivering BA to all patients with post-stroke depression and the feasibility of proceeding to a definitive trial. This study will provide information on feasibility and clinical outcomes of BA for treating post-stroke depression and its acceptability to patients, carers and therapists. The results of this study will also provide data on the feasibility of delivering the BA intervention in the National Health Service (NHS) as part of routine clinical practice.

### Objectives

The overarching aim of the BEADS trial is to explore the feasibility of a study to investigate the clinical and cost-effectiveness of behavioural activation therapy for people with post-stroke depression. The primary objective is to determine the feasibility of proceeding to a definitive trial, based on

- Feasibility of recruitment to the main trial
- Acceptability of the research procedures and measures
- Appropriateness of the baseline and outcome measures for assessing impact
- Retention of participants at outcome
- Potential value of conducting the definitive trial, based upon value of information analysis

The secondary objective is to determine the feasibility of delivering the behavioural activation therapy intervention with patients with post-stroke depression, based on

- Acceptability of behavioural activation therapy to participants, carers and therapists
- Feasibility of delivering the intervention by assistant psychologists or low-intensity psychological wellbeing practitioners under the supervision of an experienced mental health practitioner
- Documentation of ‘usual care’ using a healthcare resource use questionnaire
- Treatment fidelity of the behavioural activation therapy
- Feasibility of delivery of behavioural activation therapy within current services and within a definitive trial
- Estimate the sample size required for the main RCT

## Methods/Design

BEADS is a parallel group feasibility multicentre randomised controlled trial design, with nested qualitative research, comparing behavioural activation therapy to usual stroke care for patients with post-stroke depression.

### Setting and participants

Participants will be recruited from three centres (Sheffield, Derby, and Mansfield). Participants will be identified through NHS hospital stroke databases, community stroke team databases, hospital stroke wards, caseloads of community and acute stroke teams and the voluntary sector (stroke and aphasia groups).

Participants will be included in the study if they

1. Have a diagnosis of ischaemic or haemorrhagic stroke
2. Are aged 18 years or over
3. Are living in community settings (including nursing homes)
4. Are a minimum of 3 months and a maximum of 5 years post-stroke and
5. Are identified as depressed. Depression is defined in two ways:
  - For participants who are able to complete the Patient Health Questionnaire-9 (PHQ-9 [39]): a score of ≥10 on the PHQ-9, or;
  - For participants with communication difficulties or severe cognitive difficulties who are unable to complete the PHQ-9: a score of at least 50/100 on Visual Analog Mood Scales (VAMS) Sad item [40]

Participants will be excluded from the study if they

1. Had a diagnosis of dementia prior to the stroke (based on self-report by patient/carer)
2. Were receiving medical or psychological treatment for depression at the time at which they had their stroke (based on self-report by patient/carer)
3. Are currently receiving psychological intervention
4. Have communication difficulties that would impact on their capacity to take part in the intervention (based on assessment with the Consent Support Tool [41] for people with aphasia)
5. Have visual or hearing impairments that would impact on their capacity to take part in the intervention (based on the therapist’s discretion at baseline assessment)
6. Were unable to communicate in English prior to the stroke or do not have mental capacity to consent to take part in the trial.

The criteria are designed to identify those who would be suitable for the intervention were it to be offered within clinical practice. All reasons for patient exclusion will be recorded.

### Recruitment

The specific process for recruitment will vary according to where the participant is recruited from. Clinical teams will send invitation letters to those on the hospital or community stroke databases of discharged patients. Patients will be sent a postal pack containing a covering letter, participant information sheet, reply slip, PHQ-9, VAMS Sad and prepaid envelope. Patients who are interested in taking part will return the completed PHQ-9 and VAMS Sad with the reply slip and this will be taken as implied consent to subsequent contact by the therapist. Those who are identified as not being depressed will be contacted by the therapist to be thanked for their interest and will be informed that they are not eligible. People who score as depressed but decline to participant or are identified as not meeting the remainder of the inclusion criteria then they would be advised to contact their GP. The therapist will contact patients classified as depressed to arrange a visit to check the participant meets the remainder of the inclusion criteria, to explain the study and formally invite those who are eligible to take part. At this point, they will also obtain informed consent and complete baseline assessments.

**Fig. 1:**
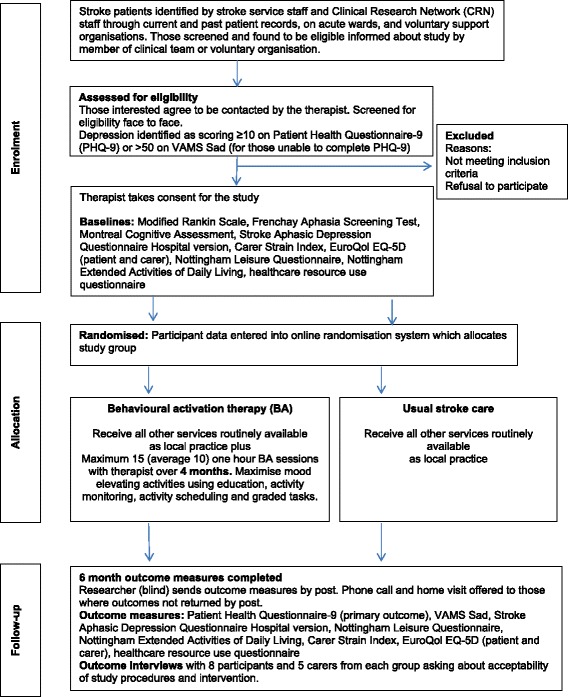
Flow of participants through the trial

Alternatively, research nurses will visit hospital stroke wards, and members of the community and acute stroke teams will be asked to identify potential participants and seek their permission to be contacted by the research team. The therapist at each centre will also seek permission to attend stroke and aphasia groups in their locality to explain the study to group members in order to identify potential participants. Willing patients will then be contacted by phone to tell them more about the research and arrange a home visit during which they will complete the screening measures, or they can request a postal recruitment pack. During the home visit (or by post), those patients who are identified as not being depressed will be thanked for their interest and will be informed that they are not eligible. For patients who are classified as depressed, the therapist will either (a) arrange a subsequent home visit or (b) continue with recruitment of the participant. Informed consent will be taken from eligible patients, who can then be entered into the study and randomised accordingly (See Fig. 1).

Self-referrals will be facilitated by advertising the study in newsletters of relevant charities and societies. Posters will also be displayed in local voluntary sector groups, libraries and local community centres so that potential participants can contact the local research team. The methods of identifying potential participants have been kept broad to allow assessment of the optimum recruitment strategy for the definitive study.

Carers of trial participants will be recruited via the trial participants during the initial home visit. They will be asked to complete the baseline and 6-month outcome assessment questionnaires.

### Randomisation and blinding

Participants will be randomised at baseline (after informed consent and baseline assessments) in equal proportions to BA or usual stroke care. Several clinical assessments will be taken at baseline: socio-demographic and stroke characteristics; Frenchay Aphasia Screening Test [42]—to assess communication difficulties; Montreal Cognitive Assessment [43]—to screen for cognitive impairment; and the Modified Rankin Scale [44]—to assess overall disability. These measures will be used to describe the baseline characteristics of the recruits.

Randomisation will be stratified by centre and will be conducted using a computer-generated pseudo-random list with random permuted blocks of varying sizes, on a remote, secure internet-based randomisation system created and hosted by the Sheffield Clinical Trials Research Unit (CTRU). Once a participant has consented to the study, the therapist will log into the randomisation system and enter basic demographic information. After this information has been entered, the allocation for that participant will then be revealed to the therapist. Access to the allocation sequence will be restricted to those with authorisation. The sequence of treatment allocations will be concealed until interventions have been assigned and recruitment, data collection and analyses are complete.

It is not possible pragmatically for the participant or therapist to be blind to the group allocation, but the researcher completing 6-month outcome assessments will be blinded and will also have had no involvement in any other aspects of the trial. The researcher will be asked to record whether or not they think they were unblinded and will also be asked to guess the group allocation. We will follow guidelines to minimise unblinding during randomised controlled trials of rehabilitation [45, 46].

### Ethical issues

The trial will be conducted in accordance with the ethical principles that have their origin in the Declaration of Helsinki, 1996; the principles of Good Clinical Practice, and the Department of Health Research Governance Framework for Health and Social Care, 2005. Participants will not be paid to participate in the trial. Ethical approval to conduct the study was granted by the National Research Ethics Service Committee for East Midlands—Leicester (ref 15/EM/0014). Local NHS Research and Development approvals have also been given for each participating centre. All trial staff and investigators will endeavour to protect the rights of the trial’s participants to privacy and informed consent and will adhere to the Data Protection Act, 1998.

### Informed consent

Written informed consent will be obtained from all participants who are able to give it. Those who lack the mental capacity to give consent are excluded from the trial. The therapists will explain the details of the trial and provide a participant information sheet, ensuring that the participant has sufficient time to consider participating or not. Informed consent to participate in the trial will be taken before participants undergo any interventions relating to the study. For patients who are physically unable to sign the form (e.g. weakness in dominant hand due to stroke) then consent will be given using a mark or line in the presence of an independent witness (who has no involvement in the trial) who will then corroborate by signing the consent form.

A significant proportion (up to 50–80 %) of the stroke population have some degree of cognitive or language impairment—aphasia. The level of support required to enable a person with aphasia to provide informed consent is dependent upon the severity and profile of the aphasia. In order to provide information in a format consistent with each individual’s language ability, a Consent Support Tool (CST) [41] will be used. The therapist will request verbal consent from the potential participant to carry out part A of the CST to determine how appropriate it is to provide the accessible information sheet. If the CST indicates that the potential participant understands less than two key written or spoken words in a sentence, they are likely to find it difficult to understand all the information required to provide informed consent. These participants will be thanked for their time but are not eligible for the study as, despite the intervention using techniques to support the inclusion of those people with reduced language or cognition, the intervention does rely on achieving understanding with support and actively participating in therapeutic communication.

The accessible information sheet will be provided to those who understand at least two key written and spoken words. This follows standard aphasia-friendly principles with one idea presented per page in short simple sentences in large font. Keywords are emboldened and each idea is represented by a pictorial image to support understanding of what the study is about. The therapists will be trained to support understanding further by reading parts of the information aloud and using supportive gestures/actions.

Once the potential participant has been given the information and had sufficient time to ask questions and discuss with family or friends, the therapist will check the individual has capacity to provide informed consent by checking that they understand the information, that they can remember what the study is about and clearly express their decision in the way in which they usually communicate (speaking, writing, using a communication aid). Participants with capacity to provide informed consent who have used the accessible information provision will be provided with an aphasia-friendly consent form and asked to initial all boxes before signing. Where stroke symptoms prevent initialling of boxes or providing written consent, the patient will use a mark or line and a relative/friend should be asked to witness the fact that the participant is consenting to the study and sign and date the consent form to confirm this on behalf of the participant.

Written informed consent will also be taken from carer and therapist participants for the outcome assessments and qualitative interviews. Should there be any subsequent amendment to the final protocol, which might affect a participant’s participation in the trial, continuing consent will be obtained using an amended consent form that will be signed by the participant.

Participants have the right to withdraw from the study at any time. Individuals removed from active participation in the intervention will not be replaced. Reason for withdrawal from the intervention, if known, will be recorded. Participants may be withdrawn from the trial either at their own request or at the discretion of the investigator. The investigator may withdraw a participant in the interest of the participant or due to a deviation from the protocol. Participants may discontinue their allocated intervention or withdraw from the study for the following reasons: withdrawal of consent; changes to their health status preventing their continued participation or failure to adhere to protocol requirements. The participants will be made aware that this will not affect their current or future care. Participation in the study does not mean that access to other services, which are part of usual care, will be compromised.

More specifically, if during the trial there is a patient allocated to the BEADS intervention who subsequently needs clinical psychology input (as per the protocol of the local service) then the BEADS therapist (AP/PWP) will discuss this with the clinical psychologist or clinical lead and the patient and will agree what is best for the patient. If it is agreed that the patient needs immediate clinical psychology input then they would be withdrawn from the BEADS intervention and they will see the clinical psychologist, or be referred to alternative provision, as appropriate to that patient. The patient will be withdrawn from the intervention but not the overall trial, i.e. we will still be able to collect outcome data from them. We will record the number of patients who we withdraw from the BEADS intervention because of a conflict with clinical services.

### Trial treatment and regimen

#### Intervention arm—behavioural activation therapy

Behavioural activation (BA) therapy is a structured and individualised treatment which aims to increase people’s level of activity, particularly the frequency of pleasant or enjoyable events, in order to improve mood. Participants randomised to receive BA will be treated at their place of residence by an assistant psychologist at two centres or low-intensity psychological wellbeing practitioner (PWP) at one centre. They will be offered a maximum of 15 sessions of BA over 4 months, with an expected average of 10 sessions. Therapy sessions will be delivered face to face on an individual basis, at the participants’ residences and will last about 1 h. A BA treatment manual was developed for CALM based on the behavioural component of CBT for depression in stroke patients [22, 24], behavioural therapy with older people [47] and guidelines on conducting therapy with people who have aphasia [35, 47, 48]. For this trial, this therapy manual will be further revised to cover BA with stroke patients who do not have aphasia and will provide examples and practical guidance relevant to all stroke patients.

The intensity and duration of therapy is based on a study of CBT with stroke patients [24] and is informed by the CALM study in which participants received an average of nine 1-h sessions over 3 months [33]. Experience and criticism of the CBT trial [24] was that therapy was too short. The trial of BA for treating depression in primary care provided 12 sessions over 3 months [49] but this was not in a stroke sample and patients with communication and/or cognitive difficulties may require a longer duration of therapy. The duration of therapy has been increased from 3 to 4 months because the CALM study showed that it was difficult to complete sessions in 3 months due to non-availability of the participant and short-term illness. Extending therapy to 4 months will also allow flexibility to provide therapy visits to support maintenance, as might be provided in clinical practice. The number of therapy sessions will vary according to the needs of the individual and their progress in therapy. The intensity of treatment will be negotiated between the therapist and the participant, based on their progress in achieving their therapy goals.

Goals set during treatment to increase enjoyable activities will be tailored to the individual. BA also includes between-session tasks to practice exercises and increase activity levels. Behavioural treatment strategies focus on maximising mood-elevating activities. The process of BA involves identifying how the person currently spends their time, identifying activities that they would enjoy doing (this may include resuming previous activities, increasing current activity levels or introducing new activities) and setting goals to increase the number of enjoyable activities.

The BEADS therapy manual presents a programme of BA delivered across 10 sessions, although the number of therapy sessions that a participant has (up to a maximum of 15) will vary according to the needs of the individual, their progress in therapy and their abilities.

- Session 1: Introducing behavioural activation therapy for depression after stroke
- Session 2: Identifying and agreeing therapy goals
- Session 3: Monitoring activity levels
- Session 4: Identifying enjoyable activities
- Session 5: Activity scheduling: enjoyable activities
- Session 6: Activity scheduling: increasing activity levels
- Session 7: Activity scheduling and increasing enjoyable activities: problem solving
- Session 8: Reviewing previous goals and setting new goals
- Session 9 Generalising behavioural activation strategies
- Session 10: Reviewing skills and making plans

Behavioural therapy techniques in the BEADS therapy manual include

Activity monitoring: Identifying how participants spend their time to assess current activity level, what activities they enjoy and when activities could be carried out. Participants are given an activity diary or timetable to complete as a between session task. The complexity of the diary will vary according to the cognitive and communication abilities of the patient and will be available in a range of formats.

Activity scheduling: Planning in advance realistic activities and goals for the participant to complete each day, which increases the likelihood that activities will be carried out. The intention is to gradually increase activities, and therefore the amount of positive reinforcement received, in order to improve mood. Activities will be set according to the abilities and goals of the individual.

Graded task assignment: Breaking a large task into smaller, manageable steps provides the opportunity to practise tasks participants find difficult. For example, for someone who wants to go shopping, they would start by going to a familiar local shop where they know people already; this would then be extended to going to a larger shop which is further away.

At two of the study sites, the therapists will be an NHS employed assistant psychologist. These are psychology graduates who work under the supervision of a clinical psychologist. At the third study site, the therapist will be a low-intensity psychological wellbeing practitioner who will have completed an accredited postgraduate certificate to qualify them as a psychological wellbeing practitioner. The therapists will attend a 2-day training workshop on BA led by an NHS consultant clinical psychologist and the chief investigator. The workshop will also include training from a speech and language therapist on communicating with stroke patients with cognitive and/or communication difficulties. Weekly clinical supervision for the therapists will be provided by a local clinical psychologist at each centre. In addition, the therapists will have a monthly teleconference with the chief investigator and NHS clinical psychologist.

#### Control arm—usual care

Participants in the usual care group will follow the current care pathway. Participants will receive all other services routinely available to them as local practice but will have no contact with the trial therapist. This group is the control arm and their care will be recorded to document usual care to inform the design of the definitive trial. Stroke survivors are admitted to hospital, usually to a stroke unit. On discharge, they may receive input from an early supported discharge team or input from a community stroke or rehabilitation team. Availability of psychological support in the community is inconsistent and likely to vary widely across the country as highlighted by the Stroke Improvement Programme [50]. The CALM trial [33] found that at the 3-month follow-up, only 14 % of participants had received mental health treatment in the past 3 months (from a mental health nurse, counsellor, psychologist or psychiatrist) and this decreased to 10 % at the 6-month follow-up. Although Improving Access to Psychological Therapies (IAPT) have extended their remit to include people with physical health problems [51, 52] the current rate of uptake by stroke patients is unknown.

Only patients not currently receiving psychological intervention are eligible to be recruited to BEADS. The provision of clinical psychology varies. Some sites have a full-time clinical psychologist providing input to both hospital and community services and other sites can access this service but do not have dedicated provision. IAPT services will consider treating stroke patients if referred, but this rarely occurs. GPs may prescribe anti-depressants. Only those with severe mental health problems are referred to psychiatrists. The content of usual care is decided locally by the clinical team as to what this will be, as per local services.

#### Intervention fidelity

To ensure the fidelity of the intervention, the content of treatment will be described and analysed. This will be achieved by video recording up to 24 intervention sessions, eight at each of the three centres. Participants and sessions will be selected iteratively using purposive sampling to represent the range of severity of depression (mild, moderate, severe from baseline scores) and across the phases of therapy (beginning, middle and end). Practices for video recording will draw upon guidance on minimising intrusiveness of the recording [53, 54]. The assessors analysing the videos will apply a customised therapy record form designed to capture a variety of key elements spanning all aspects of the intervention. Should a participant decline video recording, they will be offered audio recording instead. Participants will not be excluded from the study if they do not want to be video or audio recorded.

The therapists will also keep treatment notes for each session to summarise the content of the intervention, and to record goals set during BA and whether these were achieved. Therapists will also complete a record form of therapy content per session. The record form will quantify the content of the intervention (for example, how much time in each session was spent on different components). The record form will be based on a time sampling sheet adapted from that used in the CALM study and based on the content of the BA manual for this study. There will be triangulation between the videos, therapy record form and manual.

Qualitative interviews will be conducted with 16 participants (eight per arm), 10 carers (five per arm), and all three therapists by an independent researcher to provide a description of the acceptability of the design and procedures used in the trial and the BA intervention. All participants who participate in an interview will provide informed consent to do so. Participants will be selected iteratively using purposive sampling to represent participants from all three centres, the range of severity of depression (from baseline scores) and representation of stroke survivors with and without aphasia. All interviews will be audio recorded.

#### Patient-centred outcomes

In addition to the feasibility outcomes, the primary clinical outcome measure at 6 months is the PHQ-9 [39]. For those participants with moderate to severe language problems who are unable to complete the PHQ-9, the Visual Analog Mood Scales (VAMS) Sad item [40] will be used—this is a single-item visual analog mood measure. The number of participants unable to complete the PHQ-9 will be recorded, and the VAMS Sad will be completed with all participants so the relationship between the two measures can be explored.

The following measures will also be used to assess outcomes at 6 months:

- Stroke Aphasic Depression Questionnaire—Hospital version (observer-rated depression) [55]
- Nottingham Leisure Questionnaire (leisure activities) [56]
- Nottingham Extended Activities of Daily Living (functional outcome) [57]
- Carer Strain Index (carer-rated level of strain) [58]
- EuroQol EQ5D (health-related quality of life)—standard version [59] and picture-based version intended to be accessible for people with cognitive problems [60] for patients and carers (and proxy version)
- Healthcare resource use questionnaire

#### Sample size calculation

As a feasibility study, this is not powered for efficacy and no formal interim analyses of efficacy are planned. Rather, the sample size for a feasibility study should be adequate to estimate the uncertain critical parameters (standard deviation for continuous outcomes; consent rates, event rates, attrition rates for binary outcomes) needed to inform the design of the definitive RCT with sufficient precision. The sample size of 60 patients allows standard deviation for continuous outcomes, such as the PHQ-9 and VAMS Sad, to be estimated to within precision of approximately ±19 % of its true value (with 95 % confidence). Allowing for 15 % attrition by 6-month post-randomisation follow-up, 72 participants need to be recruited. To achieve the target sample size of 72, over the 12-month recruitment period, with three centres we need to randomise two participants per centre per month.

#### Data analysis

All trial analyses will be conducted according to an a priori statistical analysis plan that will be prepared during the early stages of the trial in agreement by the Trial Management Group, Trial Steering Committee and the Data Monitoring and Ethics Committee. Primary analysis will be conducted on the intention-to-treat population; however, exploratory analysis may be conducted excluding patients who do not comply with the protocol.

As the trial is a pragmatic parallel group, the data will be reported and presented according to the CONSORT 2010 statement [61]. As a feasibility study the main analysis will be descriptive and focus on confidence interval estimation and not formal hypothesis testing and will be guided by the Thabane et al. (2010) checklist for reporting of pilot trials [37]. Rates of consent, recruitment and follow-up by centre and by randomised group will be reported. Outcome measures will be summarised overall and by randomised group, to inform sample size estimation for the definitive trial. The data from this feasibility study will be used to estimate the consent rate, attrition rate and the variability of the continuous outcomes in the trial population and use this information to inform the sample size calculation for the definitive RCT. Study site will be treated as a covariate in an adjusted analysis where we estimate the treatment effect adjusted for baseline score and site. Since the intervention is therapist led, the data will be used to estimate the intra-cluster correlation coefficient for patients treated by the same therapist using a marginal or random effects model for the 6-month post-randomisation PHQ-9 outcome.

As part of the feasibility analysis, the effect size for the 6-month PHQ-9 outcome and the difference in mean scores (and associated 95 % confidence intervals [62]) between groups will be estimated. A marginal or random effects model will be used to allow for any clustering by therapist, with baseline PHQ-9 as a covariate to check that the likely effect is within a clinically relevant range, and this will be used as confirmation that it is worth progressing with the definitive trial. The accuracy of the cut-off of the VAMS Sad in comparison with the PHQ-9 will also be checked. This information along with the acceptability of the study design and protocol to patients and carers; the safety of the intervention; patient recruitment and consent/retention rates will enable us to determine whether or not the definitive RCT is feasible.

#### Health economic analysis

For the health economic analysis a cost-utility analysis will be undertaken from the NHS and personal social service perspective. Due to the importance of carers for patients with post-stroke depression, a supplementary analysis will be undertaken, taking a societal perspective. Costs and utilities will be estimated for individual patients using data collected at baseline and follow-up, based upon responses to EQ5D and resource use questionnaires, combined with standard cost and valuation sources [63–65]. Costs will include intervention costs and health care resource use. Questionnaires will be tested as a method for collecting resource use data and information on carer time.

Participants who do not have moderate or severe language problems will be asked to complete the standard version of the EQ5D as well as an amended picture-based version that is intended to be accessible to people with aphasia. This has not been validated but has been used in studies with similar patient populations [60, 66, 67]. Participants who do have moderate to severe language problems will be asked to complete the accessible version of the EQ5D. In addition, for participants who have carers, the carer will be asked to complete a standard EQ5D by proxy [60]. This will allow us to test alternative methods for collecting data from which to calculate quality-adjusted life years (QALYs) relevant for the patients included in the study. Utility scores based upon EQ5D responses will be calculated for patients at baseline and follow-up and QALYs will be calculated using the area under the curve defined by the scores and straight line interpolation.

Differences between costs and QALYs in the two groups will be described and the incremental cost-effectiveness ratio will be calculated. A trial-based analysis will be supplemented by an analysis using a simple decision analytic model, which will be used to estimate the cost-effectiveness of the intervention over the lifetime of the patients. This will be populated using the trial data plus information from the literature where required. Whilst this analysis will allow the estimation of lifetime cost-effectiveness and associated cost-effectiveness acceptability curves through the use of probabilistic sensitivity analysis, it is recognised that this will represent only a provisional estimate of the potential cost-effectiveness of the intervention, due to the nature of the feasibility study. The key outcome from the economic evaluation will be provided by a value of information analysis which will allow us to identify those model parameters that are the best candidates for further research [68]. This will be done by estimating expected values of perfect information for each parameter, which in essence identifies the maximum return for additional research [69].

#### Qualitative data analysis

The qualitative data will be analysed thematically, using a framework analysis approach [70] to allow us to explore both a priori and emergent themes across the dataset. The transcripts from the patient and carer interviews will be explored to understand their experiences of being recruited into the trial, the study procedures and for those in the intervention group, their experiences of the BA therapy, acceptability and any perceived impacts or benefits. The transcripts of the interviews with the therapists will be examined to understand the feasibility and acceptability of the study and intervention procedures in more depth, with a view to informing the design of any future trial and subsequent intervention.

#### Anticipated risks and benefits

This study is not an investigation of a medicinal product and entails no invasive procedures. The benefits of BA suggested by the CALM trial include improved mood [33]. No participants will have any existing treatments withdrawn. The intervention is low risk to the trial participants however, and stopping on grounds of patient safety is not anticipated. As this is a feasibility trial, it will not stop early for efficacy. The study may be stopped as a whole because of safety concerns or issues with study conduct at the discretion of the sponsor. There are no formal statistical criteria for stopping the trial early. Decisions to stop the trial early on grounds of safety or futility will be made by the Trial Steering Committee (TSC) on the basis of advice from the Data Monitoring and Ethics Committee (DMEC).

There is a risk that participants may experience some distress from being asked about their mood, but all researchers and therapists will be trained to deal with these situations. If at any point during the baseline assessment, intervention or outcome assessment the researcher or therapist is concerned about a participant, for example, severe distress or reporting feeling suicidal, then the necessary referrals will be made.

#### Suicide and suicidal intentions

The risks of suicide are inherent in the nature of the condition under scrutiny (depression), and for the purposes of this study are classed as adverse events. We will follow good clinical practice in monitoring for suicide risk during all encounters with trial participants. Where any risk to patients due to expressed thoughts of suicide is encountered, we will follow local suicide protocols for each participating site.

#### Data management

The case report form (CRF) will be used to record details at all stages of the study. CRF data will be entered at participating sites by trial staff onto an online database developed and managed by the Sheffield Clinical Trials Research Unit. Data will be stored in line with standard operating procedures. In order to help ensure good data quality, validation checks will be applied at point of entry, and comprehensive post-entry validation reports will be run regularly to generate discrepancies for site staff to investigate. Additionally, a sample of paper CRF records will be verified against the database data to identify any data entry issues.

#### Site monitoring

Site monitoring will be completed before, during and after the trial to monitor trial data quality. For example, confirmation of informed consent; source data verification (review of paper CRFs against source data); data entry verification (review of database records against paper CRFs); data storage and data transfer procedures; local quality control checks and procedures. Entries on CRFs will be verified by inspection against the source data. A sample of CRFs (at least 10 %) will be checked on a regular basis for verification of all entries made. In addition, the subsequent capture of the data on the trial database will be checked. Where corrections are required these will carry a full audit trail and justification.

#### Publication and dissemination policy

We will disseminate findings in peer-reviewed scientific journals and clinical and academic conferences, both national and international. A final report and monograph will be produced for the funder (National Institute for Health Research Health Technology Assessment). We will ensure regular dissemination to interested parties via the study website or mailing lists. A lay summary of findings for participants, service users and carers that is accessible to stroke survivors will be produced in consultation with the PPI group. An executive summary will be prepared for the Trusts where the research was conducted.

#### User and public involvement

A Public and Patient Involvement group has been formed for the study and will have input at the planning, conduct, analysis and dissemination stages of the study. A plain English summary of study progress will be provided to the group every 6 months and they will meet at regular intervals throughout the study. Information materials for participants will be developed in consultation with the group to ensure they are appropriate and accessible. Involvement will also include advice on considerations of how best to deliver the intervention to the stroke population, from the service users’ perspective; contributing to ideas on recruitment strategies. This group will also advise on the dissemination materials.

#### Trial management

Three committees have been established to govern the conduct of the study: the Trial Steering Committee (TSC), Data Monitoring and Ethics Committee (DMEC) and the Trial Management Group (TMG). The TSC will consist of an independent chair with clinical and research expertise in the topic area and three other topic experts. The TSC will meet at least every 6 months to supervise the overall conduct of the trial. The DMEC will consist of an independent chair with clinical and research expertise in the topic area, and two other topic experts, plus an independent medical statistician. The role of the DMEC is to review serious adverse events thought to be treatment-related and look at outcome data regularly during data collection. They will meet at least annually.

## Discussion

This pilot trial is designed to assess the feasibility of a definitive multicentre RCT to evaluate the clinical and cost-effectiveness of BA for treating post-stroke depression. Ethical approval was obtained on 29 January 2015. Recruitment to BEADS started in May 2015 and is planned to run until the end of April 2016.

## Abbreviations

BA, behavioural activation; BEADS, Behavioural Activation Therapy for Depression after Stroke; CALM, Communication and Low Mood; CBT, cognitive behavioural therapy; CRF, case report form; CST, Consent Support Tool; CTRU, Clinical Trials Research Unit; DMEC, Data Monitoring and Ethics Committee; IAPT, Improving Access to Psychological Therapies; PHQ-9, Patient Health Questionnaire-9; RCT, randomised controlled trial; TSC, Trial Steering Committee; VAMS, Visual Analog Mood Scales
